# A dataset of 175k stable and metastable materials calculated with the PBEsol and SCAN functionals

**DOI:** 10.1038/s41597-022-01177-w

**Published:** 2022-03-02

**Authors:** Jonathan Schmidt, Hai-Chen Wang, Tiago F. T. Cerqueira, Silvana Botti, Miguel A. L. Marques

**Affiliations:** 1 Institut für Physik, Martin-Luther-Universität Halle-Wittenberg, 06120 Halle (Saale), Germany; 2 CFisUC, Department of Physics, University of Coimbra, Rua Larga, 3004-516 Coimbra, Portugal; 3 Institut für Festkörpertheorie und -optik and European Theoretical Spectroscopy Facility, Friedrich-Schiller-Universität Jena, D-07743 Jena, Germany

**Keywords:** Theory and computation, Condensed-matter physics

## Abstract

In the past decade we have witnessed the appearance of large databases of calculated material properties. These are most often obtained with the Perdew-Burke-Ernzerhof (PBE) functional of density-functional theory, a well established and reliable technique that is by now the standard in materials science. However, there have been recent theoretical developments that allow for increased accuracy in the calculations. Here, we present a dataset of calculations for 175k crystalline materials obtained with two functionals: geometry optimizations are performed with PBE for solids (PBEsol) that yields consistently better geometries than the PBE functional, and energies are obtained from PBEsol and from SCAN single-point calculations at the PBEsol geometry. Our results provide an accurate overview of the landscape of stable (and nearly stable) materials, and as such can be used for reliable predictions of novel compounds. They can also be used for training machine learning models, or even for the comparison and benchmark of PBE, PBEsol, and SCAN.

## Abstract (summary)

**Table:** 

Measurement(s)	optimized geometry (PBESol) • total energy (PBESol, Scan) • bandgap (PBESol, Scan)
Technology Type(s)	Density functional theory (VASP)
Factor Type(s)	Exchange correlation functional • Crystal structure

## Background & Summary

The search for new materials remains one of the most important quests but, unfortunately, also one of the great challenges of materials science. Nowadays, data-driven searching strategies have become the most cost-effective methods to tackle this problem, and the fastest way of finding new materials or study their properties are computational high-throughput searches. After years of data accumulation, there are millions of calculations of materials available in open databases^1,2^ that are used as an invaluable reservoir to select and filter promising candidates for further experimental synthesis and characterization.

These high-throughput studies in solid-state material science^3–6^ have broadened the exploration of the vast chemical space, while plenty of works have successfully found and predicted promising materials for technological applications. However, nearly all high throughput searches rely on the use of density functional theory (DFT) within the Perdew-Burke-Ernzerhof (PBE) approximation to the exchange-correlation functional^7^. This is a well established and reliable approach that earned its place as the standard technique in solid-state research. However, the PBE functional is now over 25 years old, and more recent (and accurate) functional have by now been proposed in the literature. For example, the Armiento-Mattson 2005^8^ or the PBE for solids^9^ functionals consistently lead to superior geometries^10,11^, while the SCAN meta-generalized gradient approximation^12^ yields formation energies that are on average better by a factor of two than the PBE^13^. Unfortunately, and in stark contrast with the abundance of PBE data, there are no available comparable large scale datasets calculated with these improved functionals.

There are a number of other databases that use either higher accuracy methods, like G_0_ W_0_, or apply density-functionals different from PBE. For example, we can mention the Computational 2D Materials Database^14,15^ that provides a dataset of 4000 2D materials calculated with HSE, G_0_ W_0_, RPA and the Bethe-Salpeter equation, while JARVIS contains a large number of calculations with vdW corrections using OptB88vdW^16,17^ and performed with the modified Becke-Johnson potential^18–20^.

In a previous work^21^ we combined data from the AFLOW database^1^, the Materials Project^2^ and from our own group to create a rather complete convex hull of thermodynamic stability at the PBE level. The details of the selection of the dataset can be found in ref. ^21^. Specifically, we selected all materials that were calculated with the same functional, pseudopotential, as well as *U*-parameters used in the Materials Project. We removed duplicates, i.e. entries with the same space group, composition and total energy (rounded to the 4th digit). Here we determined the space group using pymatgen with the “symprec” keyword set to 0.1. From the AFLOW database we further removed all prototypes labeled “_DEVIL_PROTOTYPES_” and all other combinations of prototypes and pseudopotentials that are noted as ill-converged in the code of ref. ^22^. As AFLOW is still known to contain outliers^22^, we also removed them from the calculation of the hull following a strategy similar to the one explained in ref. ^22^. We used the total energies of all the remaining structures to calculate the convex hulls applying the corrections from the materials project workflow to the energies.

From this dataset^23^ we selected around 175k compounds that were either stable (i.e., on the convex hull), or close to stable (within 100 meV/atom of the hull). These were then reoptimized with PBEsol^9^. Finally, the total energies were reevaluated with SCAN^12^ to create highly accurate formation energies and convex hulls.

## Methods

Our starting point was the dataset used in the machine learning study of ref. ^21^. This included PBE calculations stemming from the Materials Project database^2^, AFLOW^1^, and our own calculations. These were then filtered to obtain a homogeneous set in what regards the calculation parameters, leading to a dataset containing more than two million compounds. We then constructed the convex hull of thermodynamic stability and extracted entries that were either on the hull or within 0.1 eV/atom^24,25^. The reason for the choice of this cutoff was twofold. First, its value is still below the average error in the formation energies calculated with PBE^26,27^, but is larger than the estimated error in the distances to the convex hull^28,29^. As such, the compounds that were misidentified by the PBE as thermodynamically unstable are likely to be included in the set. Second, there are a number of materials that are metastable, but experimentally accessible (for example for compositions that have more than one polymorph). We can reasonably expect that the cutoff allows for the inclusion of the majority of these cases. We also eliminated materials with unit cells that were too large for our computational resources, leading to a final amount of ~175k compounds.

All calculations were performed using density-functional theory, within the projector augmented wave method (PAW)^30^ as implemented in the Vienna *ab initio* simulation package (VASP)^31,32^. We used the PAW setups shipped with version 5.4 of vasp that include information on the kinetic energy density of the core electrons. We mostly followed the recommendations of the Materials Project for the choice of the pseudopotentials. The exception was Cs, for which we used an improved pseudopotential generated by the vasp developers, as the stock PAW setup often led to negative densities during the self-consistent cycle, crashing the calculation. All calculations were performed taking into account spin-polarization, and started from a ferromagnetic configuration (as in the large majority of high-throughput studies). This most likely leads to an incorrect spin configuration for antiferromagnetic systems, resulting in an energy higher than the true groud-state. However, it is well known that in most cases magnetic exchange energies are rather small, so the error in the total energy is limited to a few tens of meV/atom. Methfessel-Paxton order one smearing with a width of 0.2 eV was applied in the integration of the Brillouin zone.

The structures were optimized using the PBEsol^9^ approximation, the “High” precision keyword of vasp, and a Γ-centered k-point grid with 2000 k-points per reciprocal atom, until the forces on the atoms were below 5 meV/Å. To detect the structures that required re-optimization we checked that the the absolute value of the forces (in meV/Å) and the individual components of the stress tensor (in meV/Å^3^), calculated with PBEsol with stricter convergence criteria (520 eV for the wave-function cutoff and 8000 k-points per reciprocal atom), remained smaller than 0.05. If it was not the case, we increased both the energy cutoff and the number of k-points and performed again the geometry optimization. Around 3% of all structures required this further calculation step. We note that as we had already a reasonable starting point, specifically the PBE geometry, the geometry optimization required a relatively small number of steps in most cases.

A final energy evaluation with the SCAN meta-GGA functional was performed with a cutoff of 520 eV, 8000 k-points per reciprocal atom, and including the non-spherical contributions from the gradient corrections inside the PAW spheres. As expected, SCAN calculations were much more unstable than PBEsol, due to the well known numerical instabilities of this functional^33,34^, which led to a much lower average convergence rate. In any case, we succeeded in converging nearly all calculations. In total, the calculations presented here required around 10 million CPU hours.

## Data Records

The output files of vasp were collected and processed with the pymatgen library^35^. Each final data record consisted of a *ComputedStructureEntry* that included the chemical composition, the total energy, and the detailed crystal structure of the entry. Two files, containing all entries for each functional, can be freely downloaded from the Materials Cloud repository^36^ and can be loaded trivially using the json module of python. For convenience, we also provide a summary of the data in tabulated form, that include the fields listed in Table 1.

**Table 1:** Fields included in the summary of the data in tabulated form.

Composition	Chemical composition of each material.
Number of sites	Number of atoms in the unit cell.
*E*_PBE_	Total energy per atom calculated with PBE (extracted from the primary dataset)
*E*_PBEsol_	Total energy per atom calculated with PBEsol.
*E*_SCAN_	Total energy per atom calculated with SCAN.
*V*_PBE_	Volume per atom of the PBE unit cell (extracted from the primary dataset).
*V*_PBEsol_	Volume per atom of the PBEsol unit cell.
Gap_PBEsol_	Band gap calculated with PBEsol.
Gap_SCAN_	Band gap calculated with SCAN.
*M*_PBEsol_	Total magnetic moment per atom calculated with PBEsol.
*M*_SCAN_	Total magnetic moment per atom calculated with SCAN.
*S*_SCAN_	The diagonal elements of the stress tensor calculated with SCAN.

**Fig. 1:**
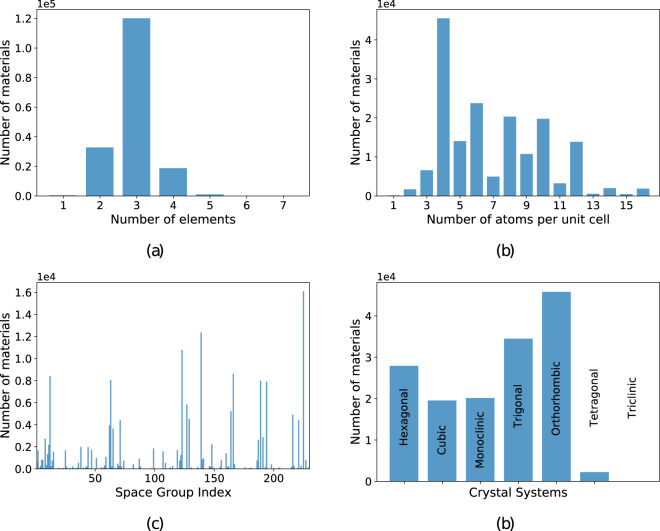
Distribution of (**a**) number of different chemical elements per unit cell, (**b**) number of atoms per unit cell, (**c**) index of space groups, and (**d**) crystal systems for all the materials in our dataset.

In panel a of Fig. 1 we plot the histogram of the number of different chemical elements in our materials. We can clearly see that the dataset is dominated by ternary compounds, followed by binary and quaternary. Relatively few multinary materials with more than 4 different chemical elements are present. We can understand this distribution by considering that the number of permutations increases very rapidly with the number of chemical elements, explaining, e.g., why we have many more binaries than elementary substances or ternary than binary compounds. However, we have to keep in mind that the complexity of the unit cells also increases and that most of the systematic high-throughput DFT studies were performed for ternary systems^37–42^. This can also be seen in panel (b) of Fig. 1 where we show a histogram of the number of atoms in the primitive unit cell. The distribution is dominated by a peak centered around five atoms per unit cell, arising from the materials stemming from the high-throughput studies of AFLOW^1^ and from our group^3,37,38^. Obviously, we do not expect that the true distribution of all possible thermodynamically stable and metastable materials follows the behavior depicted in Fig. 1. Finally, in panels (c) and (d) of the same figure, we plot an histogram of the number of materials as a function of the space group index and of the crystal system. We see that the most represented systems are the trigonal and orthorhombic, while the tetragonal and triclinic are the least represented.

**Fig. 2:**
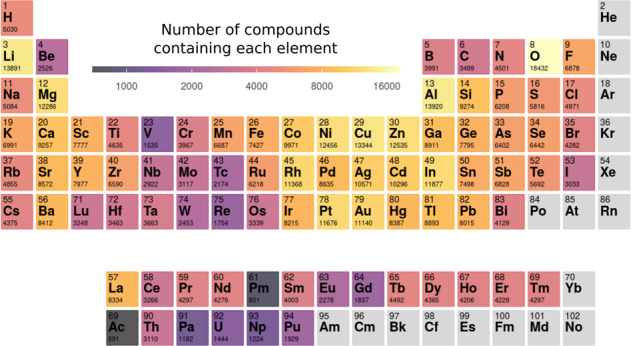
Periodic table depicting the chemical elements present in our dataset. The number beneath the chemical symbol is the number of materials present in the dataset that contain the given element.

In Fig. 2 we show the distribution of chemical elements for the calculated structures. We considered all elements except noble gases up to bismuth as well as most lanthanides, and actinides up to plutonium. We can observe some obvious trends. Not surprisingly, the most common element is oxygen due to the abundance of oxides in our planet and their stability in our oxygen-rich atmosphere. The remaining chalcogens (S, Se, Te) are all equally represented, which is probably an indication of their chemical similarity. For the halogen family we see a decreasing number of materials following the decrease of the electronegativity, with iodine compounds being half as abundant as fluorides. For the pnictogens we witness exactly the opposite behavior, with much fewer nitrides than compounds containing antimony. This can be understood from the high chemical stability of the N_2_ molecule and the rather high nominal oxidation state of nitrogen (−3) that hinders the synthesis of nitrides. Finally carbon-containing materials are rather scarce due to the absence of organic compounds in our dataset.

From the metals, the two with the highest number of compounds are aluminum and lithium. The former is within a cluster of highly represented chemical elements (such as nickel, copper, zinc, or indium). The exception in this region of the periodic table is gallium, in spite of its importance in many semiconductors used in electronics and optoelectronics. Lithium compounds, on the other hand, are much more common in our dataset than materials containing any other alkali element, which might be explained by the popularity of studies in lithium compounds in view of their application in battery technologies. Interestingly, in contrast to the other alkali earth elements, beryllium appears in relatively few compounds, probably due to its high toxicity. Another region that exhibits relatively few compounds is the one centered in vanadium and that includes rhenium, hafnium, niobium, etc. We can observe a continuity in the values in this region that might indicate that these chemical elements, often exhibiting the very high oxidation numbers of +4, +5 or +6, have more difficulty producing stable compounds than other metals with lower oxidation numbers. Finally, the least represented groups are unsurprisingly the lanthanides and the actinides, showing how little we know the chemistry of these chemical elements that are essential in a multitude of technologies, such as in hard magnets or in the storage of nuclear waste.

It is also interesting to look at the structural diversity in our dataset. With that objective, we used pymatgen to divide our structures into groups according to structural similarity. In total, our data turned out to contain 24706 prototypes of 4557 different generic compositions. However, and as expected, the distribution of compounds among these prototypes was rather unbalanced. For example, 15399 prototypes only appeared once in the dataset and 2412 only twice. On the other hand, most materials belong to just a few prototypes. The most common was by far the Heusler family of compounds, with almost 12 000 compositions, followed by the double perovskite family with more than 5000 elements. Of course, these numbers reflect not only the chemical stability and size of each one of the families, but also the interest of the community for these compounds.

## Technical Validation

**Fig. 3:**
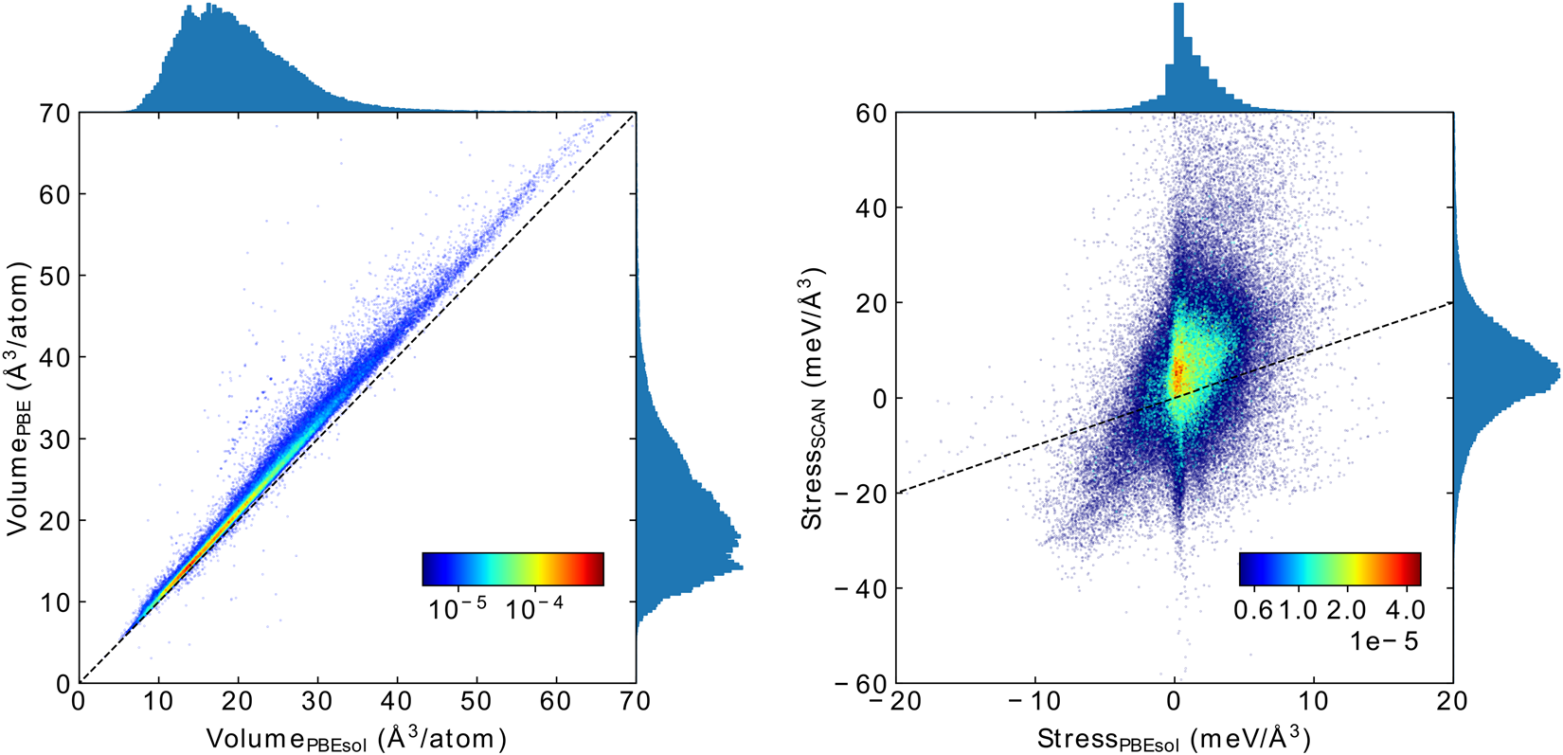
Left: Distribution and scatter plots of volumes per atom calculated with PBE (from the primary data) and PBEsol functionals. The width of the bins is 0.35 Å^3^/atom. Right: distribution and scatter plots of the diagonal components of the stress tensor calculated with PBEsol and SCAN at PBEsol geometries. The width of the bins is 0.6 meV/Å^3^.

In the left panel of Fig. 3 we plot the distribution of the volumes per atom with the PBE (obtained from the primary data) and PBEsol. Both curves look similar, with a peak at around 15 Å^3^ and skewed towards larger volumes. We can also observe that the PBE data is shifted toward larger volumes with respect to PBEsol. This is due to the well known underbinding of the PBE^43^ that leads to lattice constants that are larger than their experimental values by 2–3% (and consequently volumes that are overestimated by ~10%). This underbinding is almost totally corrected by PBEsol, yielding smaller volumes in much better agreement with experiment. In the right panel of the same figure we plot a histogram of the three diagonal components of the stress tensor calculated with SCAN at the PBEsol geometry. We can see that the calculations yield rather small stresses, showing that the structures are close to mechanical equilibrium. This is expected because SCAN, like PBEsol, yields high quality geometries in good agreement with experiment^10,44^. This also validates our pragmatic approach of using a single point SCAN calculation at the PBEsol geometries.

**Fig. 4:**
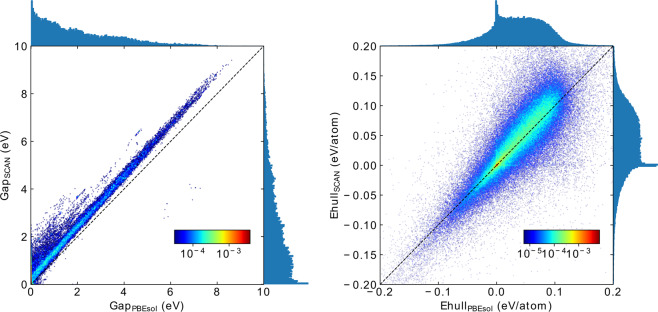
Left: Distribution and scatter plots of the (in)direct band gaps calculated with PBEsol and SCAN at the PBEsol geometry. The width of the bins is 0.1 eV. Right: Distribution and scatter plots of the distances to the convex hull calculated with PBEsol and SCAN at PBEsol geometries. The corresponding hulls contain 40246 and 38692 materials. The width of the bins is 2 meV/atom.

In Fig. 4 we show the distribution of (indirect) band gaps calculated with both PBEsol and SCAN. As expected, the curves decay monotonically with the value of the gap, and exhibit a fat tail that extends beyond 10 eV. It is known that PBEsol, as well as PBE, strongly underestimate the value of the band gaps by essentially a factor of two, leading to mean absolute percentage errors bordering the 50%^45,46^ with respect to experiment. This is to some extent corrected by SCAN, that increases consistently the band gaps leading to a mean absolute percentage error of around 40%^45,46^. This increase is evident from Fig. 4, where the SCAN band gap distribution is shifted to the right with respect to PBEsol.

Finally, we performed an analysis of the convex hulls obtained with PBE, PBEsol, and SCAN. The hulls contained 36985, 40246 and 38692 materials respectively. These differences are expected, and mostly stem from relatively small changes in the formation energy for compounds that were close to the hull. The distributions of the distances to the convex hull of thermodynamic stability are plotted in the right panel of Fig. 4. For this plot, we always removed the compound under study from the hull, allowing therefore for negative distances. Although this allows for a better interpretation of the results, we should remember that technically speaking all compounds with negative distances should be placed strictly at zero. Both distributions grow fast for negative distances to the hull, having a marked peak at zero. The main contributors to this behavior are the experimental compounds. The number of materials with positive distance to the hull is relatively constant, until reaching our cutoff at ±0.2 eV/atom. This cutoff is obviously smeared for the PBEsol and SCAN calculations.

**Fig. 5:**
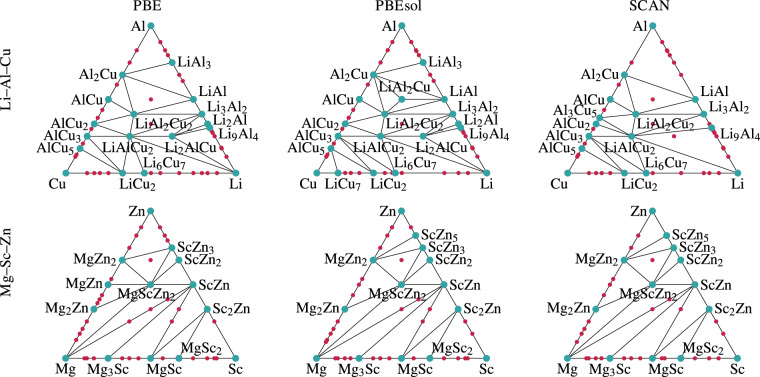
Ternary phase diagrams of the Li–Al–Cu (upper panel) and Mg–Sc–Zn (lower panel) systems, calculated with the PBE (left), PBEsol (middle) and SCAN (right). The blue points indicate compositions on the convex hull, while red points denote materials that are within 50 meV/atom from the hull.

As an illustration, we plot in Fig. 5 the ternary phase diagrams of Li–Al–Cu and Mg–Sc–Zn. We see that the three functionals agree to a large extent on which are the stable materials. However, some differences are also clear. For example, MgZn is stable with the PBE functional but not with PBEsol or SCAN, or LiAl3 is unstable with SCAN but stable with the other two functionals. In any case, compounds that are stable with one functional do appear stable, or at least metastable with the other functionals. Of course, SCAN is the most accurate of the three functionals in what concerns formation energies and distances to the convex hull, so the SCAN diagrams should on average have the highest accuracy.

## Usage Notes

The data can be downloaded from the Materials Cloud repository^36^. The energies, compositions, and structures for each material are formatted as *ComputedStructureEntries* and stored as compressed json files. They can therefore be trivially loaded in python and analyzed with pymatgen. We note that we used version v2019.10.2 of pymatgen, but the data should be compatible with other versions.

## Data Availability

All data can be easily processed with publicly available tools such as json and pymatgen^35^. An example usage is provided with the data. The dataset was generated with VASP, the bash and python scripts to generate input files or manage the output files can be downloaded from github repository: https://github.com/hyllios/utils/tree/main/ht_pd_scan.
